# A Rare Culprit of Infective Endocarditis in an IV Drug User: *Burkholderia cepacia*

**DOI:** 10.1155/2019/6403943

**Published:** 2019-04-10

**Authors:** Christopher Nnaoma, Ogechukwu Chika-Nwosuh, Christoph Sossou

**Affiliations:** Department of Medicine, Newark Beth Israel Medical Center, Robert Wood Johnson Barnabas Health, Newark, NJ, USA

## Abstract

Infective endocarditis (IE) is an infection of the cardiac native or prosthetic valves typically caused by *Staphylococcus aureus*, viridans streptococci group, and coagulase-negative staphylococci. Risk factors include congenital heart disease, structural and valvular heart disease, implantation of prosthetic heart valves, and intravenous (IV) drug abuse. IE caused by organisms such as *Burkholderia cepacia* is rarely seen. We herein present a case of a patient with a history of IV drug abuse previously treated for *Staphylococcus aureus* IE with newly diagnosed IE secondary to *B. cepacia*. He was taken to the operating room for mitral valve replacement after an echocardiogram revealed severe mitral regurgitation. He was successfully treated with antibiotics. After 2 months, at follow-up, the patient remained free from mechanical valve-related events, had no new occurrences of fever, and had no other symptoms of infection. He reported good exercise tolerance.

## 1. Introduction

*Burkholderia cepacia* is a group of catalase-producing, non-lactose-fermenting, Gram-negative bacteria composed of at least 20 different species [1]. *B. cepacia* is an opportunistic human pathogen affecting mostly patients with cystic fibrosis and chronic granulomatous disease [2]. Infection in immunocompetent individuals is uncommon, and *B. cepacia* has increasingly been recognized as an important pathogen of IE among intravenous drug abusers (IVDAs). It is known to be resistant to many antibacterial agents [2]. However, treatment can be conservative, requiring antibiotics and/or surgical intervention [2]. Due to its rarity in causing endocarditis, standard therapeutic options to guide clinicians' decision are lacking.

## 2. Case Presentation

A 32-year-old male with a history of intravenous drug abuse had infective endocarditis treated for 6 weeks with appropriate antibiotics and mitral valve repair and annuloplasty due to severe mitral regurgitation (MR) and tricuspid regurgitation (TR) 9 months ago. At that time, he presented to the ER for fevers, poor appetite, and lethargy which was gradually worsening over a week duration. On presentation, he was found to be febrile with a temperature of 102°F. Physical examination was pertinent for a systolic murmur best heard at the apex beat and tachycardia of 118 beats per minute. Lab examinations were notable for a WBC of 15 × 10^3^/mcl, a hemoglobin (Hb) level of 9.1 g/dl, and a lactic acid level of 3.2 mg/dl. Blood culture was done, and the patient was started on antibiotics with vancomycin and piperacillin/tazobactam. Blood culture grew Gram-positive cocci in pairs, which were later identified as methicillin-resistant *Staphylococcus aureus*. He was continued on vancomycin. Transthoracic echocardiography revealed vegetation in the mitral valve with severe MR and TR; this was confirmed with transesophageal echocardiography. He was subsequently taken to the OR for mitral valve repair and annuloplasty. The postsurgery period was uneventful, and he improved clinically and was discharged to complete a 6-week course of vancomycin.

The patient re-presented 9 months later to the emergency room with a history of shortness of breath on exertion and fatigue. He did report a history of recent intravenous drug use after completion of antibiotics for IE and reuse of needles after washing them. On physical examination, he was febrile with a temperature of 100.7°F and tachypneic with a respiratory rate of 27 breaths per minute. Lungs were clear to auscultation, and no jugular venous reflux, no pedal edema, and no skin lesions were noted. A 4/6 systolic murmur was noted. Lab examinations were pertinent for a hemoglobin level of 8.3 g/dl, a WBC of 12.0 × 10^3^/mcl, and a creatinine level of 1.5 mg/dl. Blood culture was done, and the patient was started on antibiotics with vancomycin and cefepime. Transesophageal echocardiogram showed severe mitral regurgitation (MR) status after mitral annuloplasty and a posterior directed eccentric MR jet. The MR is mostly from the P3/A3 area with rudimentary posterior leaflets and relatively A2 and A3 prolapse. A mildly thickened tricuspid valve with severe tricuspid regurgitation (TR) was also seen. No vegetations or abscesses were noted. Blood cultures grew back positive for *B. cepacia* in one bottle.

Given this, the patient was taken to the operating room for repeat sternotomy 72 hours after admission, mitral valve replacement with a St. Jude Medical mechanical mitral valve prosthesis of 27 mm, and tricuspid valve annuloplasty with a 30 mm ring. The postoperative course was uneventful. The patient remained afebrile with a normal blood cell count. The pathology report of the specimen (native heart valve) was positive for fibrous tissue and fibrinous material with few inflammatory cells. Cultures of the heart valve grew *Burkholderia cepacia* which was sensitive to trimethoprim/sulfamethoxazole, meropenem, levofloxacin, and ceftazidime. Based on Duke's criteria, the patient was diagnosed with IE.

The patient was started on treatment with intravenous levofloxacin 500 mg daily and was discharged after one week to complete 6 weeks of intravenous antibiotics at a rehabilitation center. Repeat blood cultures prior to discharge were negative for any organism. He was started on anticoagulation with warfarin and discharged to a rehabilitation center.

After 2 months, at follow-up, the patient remained free from mechanical valve-related events, had no new occurrences of fever, and had no other symptoms of infection. He reported good exercise tolerance. He denied any drug use since discharge.

## 3. Discussion

*B. cepacia* was first discovered by Walter Burkholder in 1949 as the cause of onion skin rot (hence named *Burkholderia*) and first described as a human pathogen in the 1950s (https://en.wikipedia.org/wiki/Burkholderia_cepacia_complex) [1]. *Burkholderia cepacia* is a Gram-negative, aerobic, non-spore-forming bacillus that has been described as an important pathogen in particular subgroups of patients (cystic fibrosis and granulomatous disease) [1]. It was first isolated from patients with cystic fibrosis (CF) in 1977 when it was known as *Pseudomonas cepacia* [3]. It has a low virulence and is able to survive neutrophil attack [4]. This is possible because certain species of *Burkholderia* produce extracellular polysaccharides (EPSs); this unique property and structure of EPSs may be used in a diagnostic approach for these bacteria. In study [5], it represents a very important nosocomial infection.

*Burkholderia cepacia* is an intracellular parasite with poorly defined pathogenicity, which seems to determine the activation of TNF-mediated inflammation or bacteremia, causing, in rare cases, sepsis-like manifestations; infection with this pathogen is associated with a high rate of in-hospital mortality (33%) [2]. The bacillus is an opportunistic microorganism that can invade the respiratory epithelium. The transmission mode involves horizontal transmission; hence, patients should be placed on isolation as done in our study. *B. cepacia* can also be a contaminant from the hospital water system. Therefore, we recommend thorough hand washing or use of hand sanitizers by hospital staff and also screening the hospital water system for the organism. It is commonly found in water and soil. In rare cases, it is responsible for endocarditis, especially in patients with a history of heroin abuse [6]. It was speculated that the source of infection in our patient may have been due to repeated use of the same needle cleaned with possibly contaminated water. *B. cepacia* cannot be ignored even when contamination is mooted but not confirmed in endocarditis. We recommend treating the organism and repeat blood cultures.

In our case, prior to surgery, the patient was counselled extensively about the need to stop illicit drug use as this will be a serious problem given valve replacement. He agreed to check into an addiction treatment center which he was discharged to.

Anatomical signs of infective endocarditis in the mitral position include valve dysfunction, paravalvular leaks, and annular abscesses. In particular, the incidence of paravalvular leaks (PVLs) is estimated at 2–17%: they can be asymptomatic conditions that do not always require treatment or can cause hemolysis and heart failure [7].

Diagnosis of *B. cepacia* involves culturing the bacteria from clinical specimens, such as sputum, blood, or tissue samples as done in our patient. The organism is usually cultured in *Burkholderia cepacia* agar (BC agar) or oxidation-fermentation polymyxin-bacitracin-lactose (OFPBL) agar.

Treatment is usually with IV antibiotics such as ceftazidime, doxycycline, piperacillin, meropenem, or trimethoprim/sulfamethoxazole (Bactrim). The drug of choice for *B. cepacia* infection is Bactrim. However, our patient was treated with levofloxacin, for 6 weeks owing to the side effect profile of long-term use of Bactrim and our patient's renal function.

Given the organism's high resistance to most antibiotics, the choice of antibiotics should be guided by sensitivity testing. Antibiotics should be continued for 6-week duration as recommended for infective endocarditis.

## 4. Conclusion

*B. cepacia* is a rare cause of IE and can be a contaminant. However, this infection cannot be ignored especially when isolated from the heart valve as in our patient. We recommend (1) patient isolation, (2) testing the hospital water system, and (3) treatment with appropriate antibiotics based on the sensitivity and repeat blood cultures to ensure clearance of this organism from the blood.
